# The alphaherpesvirus gE/gI glycoprotein complex and proteases jointly orchestrate invasion across the host’s upper respiratory epithelial barrier

**DOI:** 10.1128/mbio.01873-24

**Published:** 2024-10-09

**Authors:** E. Van Crombrugge, X. Ren, S. Glorieux, I. Zarak, W. Van den Broeck, C. Bachert, N. Zhang, T. Van Zele, D. Kim, G. A. Smith, K. Laval, H. Nauwynck

**Affiliations:** 1 Department of Translational Physiology, Infectiology and Public Health, Faculty of Veterinary Medicine, Laboratory of Virology, Ghent University, Merelbeke, Belgium; 2 Center for Human Body Material, Faculty of Medicine and Health Sciences, Ghent University, Ghent, Belgium; 3 Department of Morphology, Imaging, Orthopedics, Rehabilitation and Nutrition, Faculty of Veterinary Medicine, Ghent University, Merelbeke, Belgium; 4 Department of Otorhinolaryngology – Head and Neck Surgery, University Hospital of Münster, Münster, Germany; 5 Department of Head and Skin, Upper Airways Research Laboratory, Faculty of Medicine and Health Sciences, Ghent University, Ghent, Belgium; 6 Department of Microbiology-Immunology, Northwestern University, Feinberg School of Medicine, Chicago, Illinois, USA; University of Florida College of Public Health and Health Professions, Gainesville, Florida, USA

**Keywords:** alphaherpesvirus, upper respiratory tract, pathogenesis, mucosa invasion, gE/gI complex, proteases, urokinase plasminogen activator

## Abstract

Alphaherpesviruses, including herpes simplex virus type 1 (HSV-1), pseudorabies virus (PRV), and bovine herpesvirus type 1 (BoHV-1), are significant pathogens affecting humans and animals. These viruses penetrate the upper respiratory tract mucosa, yet the mechanisms facilitating this invasion are not fully understood. This study investigates the role of the gE/gI glycoprotein complex and proteases in mucosal invasion by these viruses. Using species-specific respiratory mucosal explants, we observed that the removal of extracellular calcium disrupts epithelial junction integrity, enhancing viral infection across all viruses and suggesting a common mechanism of targeting a basolaterally located receptor. PRV exhibited significantly faster replication and deeper invasion compared to HSV-1 and BoHV-1. The gE glycoprotein was consistently polarized at the basement membrane across all viruses, indicating a critical role in the process of viral entry and subsequent spread through the epithelium. In this context, “infection” refers to the virus’s attachment to its cell-surface receptor, entry into the cell, and completion of the viral life cycle, culminating in the production of progeny virions. Notably, in gE/gI null mutants of PRV and HSV-1, while the infection was not abortive and the viral life cycle was completed, the infection was delayed, and the invasion into the deeper layers of the epithelium and underlying mucosa was significantly reduced. In BoHV-1 mutants, this effect was even more pronounced, with infection restricted to the apical cells, failing to progress to the basal cells. In addition, PRV and HSV-1 invasion involved serine protease activity, unlike BoHV-1, which correlates with its slower invasion pace. Notably, the protease facilitating PRV invasion was identified as a urokinase plasminogen activator (uPA), while the specific protease for HSV-1 remains unidentified. These findings highlight the critical roles of the gE/gI complex and proteases in alphaherpesvirus pathogenesis, offering potential targets for therapeutic intervention.

**IMPORTANCE:**

Herpes simplex virus type 1 (HSV-1) infections are a worldwide issue. More than three billion people are infected with HSV-1 globally. Although most infections with HSV-1 occur subclinically, severe symptoms and complications are numerous and can be life-threatening. Complications include encephalitis and blindness. Recently, HSV-1 infections have been associated with the development of Alzheimer's Disease. To date, no effective vaccines against HSV-1 are on the market. Pseudorabies virus (PRV) and bovine herpesvirus type 1 (BoHV-1) are two alphaherpesviruses of major veterinary importance. Although efforts have been made to eradicate these viruses from livestock animals, clinical problems still occur, resulting in great economic losses for farmers. It is evident that new insights into the pathogenesis of alphaherpesviruses are needed, to develop effective treatments and novel preventive therapies.

## INTRODUCTION

Alphaherpesviruses are DNA viruses within the *Herpesviridae* family, which can infect many mammalian species. Among them, herpes simplex virus type 1 (HSV-1) is a well-known pathogen that commonly causes cold sores (herpes labialis). HSV-1 infection is widespread with around 45%–97% seropositivity worldwide (1). In neonates and immunocompromised individuals, infection with HSV-1 can have more severe consequences and can lead to encephalitis and death (2). To date, there are no vaccines available against HSV-1 infection. Recently, some evidence suggested a causal role of HSV-1 in the etiology of Alzheimer’s disease (AD) (3–7). Therefore, there is an urgent need to develop a new effective vaccine as vaccination may be a potential measure to prevent dementia. In animals, pseudorabies virus (PRV) and bovine herpesvirus type 1 (BoHV-1) are two alphaherpesviruses of great veterinary importance. PRV is the causative agent of Aujeszky’s disease in swine. The virus can cause respiratory disease, neurological disorders, and abortion in swine (8). While PRV is mainly associated with rhinitis and pharyngitis in adult pigs, the mortality in neonates and suckling pigs is close to 100% and central nervous system symptoms often precede death of the animals (9). PRV can also cause 100% mortality in many other animal species, such as cattle, sheep, goats, dogs, and cats (10). Humans are generally not susceptible to PRV infection. However, rare cases of PRV transmission to humans have been reported, usually through direct contact with infected pigs or tissues (11, 12). Over the last three decades, PRV has been eradicated in the United States and several member states of Europe using a combination of vaccination and culling. Vaccination was performed with a marker vaccine, in which the viral glycoprotein E (gE) gene was deleted (13). However, this fact does not make PRV research redundant. First, significant genetic and functional homologies exist between alphaherpesviruses, rendering PRV extremely interesting for comparative molecular virology. Indeed, PRV is frequently used as a model to study the biology of alphaherpesviruses, such as HSV-1. For instance, molecular biologists use PRV as a model system to dissect the mechanisms of alphaherpesvirus invasion and spread *in vitro* and *in vivo* (14, 15). Second, novel strains of PRV have been detected recently, which have emerged from Bartha-K61-vaccinated pig farms in China (16, 17). Since the Bartha-K61 vaccine cannot give sufficient virological protection against these new PRV variants, these strains continue to spread and are causing considerable clinical problems in pig farms in Northern, Eastern, and Southern China, leading to substantial economic losses (18). In contrast to PRV, BoHV-1 does not have a broad host tropism. The virus is known to only infect ruminants and cause clinical signs primarily in cattle (19). The disease manifestation of BoHV-1 is also more limited than that of PRV. While historically the virus primarily affected the reproductive system, causing balanoposthitis in bulls and pustular vulvovaginitis in cows, current circulating strains are prone to respiratory tropism (20). Consequently, it plays an important role in the development of respiratory disease complexes in cattle, also known as “shipping fever,” and BoHV-1-induced respiratory disease is also commonly referred to as “infectious bovine rhinotracheitis” (IBR) (21). Using a similar strategy as for PRV, multiple countries in Europe and the United States are attempting to eradicate BoHV-1 from domestic livestock (22, 23). However, the virus may remain latently present in some herds, leading to breakthroughs. Any farm or facility (holding) with latently infected animals remains a potential source of infection for BoHV-1-free farms (24–27).

A common portal of entry for alphaherpesviruses is the upper respiratory tract (URT). Here, primary replication occurs in the epithelial cells. To date, the identity of the cellular receptor in epithelial cells remains unknown. Interestingly, it has been recently shown that equine herpesvirus type 1 (EHV-1) targets a basolaterally located receptor in the horse respiratory epithelium for efficient infection of the URT (28). After initial infection, alphaherpesviruses spread throughout the epithelium in a plaque-wise manner and then cross the basement membrane (BM). While EHV-1 is known to target diapedesis monocytic cells and T-lymphocytes to overcome the BM, PRV, and BoHV-1 directly cross this barrier and infect fibroblasts in the lamina propria (29–32). Afterward, the viruses reach the bloodstream and/or the trigeminal ganglion (TG), where they may go into latency (33, 34). PRV can initiate a cell-free and cell-associated viremia in peripheral blood mononuclear cells (mainly monocytes) (35). This strategy allows the virus to spread efficiently to secondary target organs, such as the pregnant uterus. PRV infection of the pregnant uterus can therefore lead to abortion, even in vaccinated sows (9). BoHV-1 infection of blood monocytes is rare, but some cases of BoHV-1-induced abortion have been documented in the past (20). So far, HSV-1-induced viremia has been reported rarely. Upon stress-induced reactivation or immunosuppression, alphaherpesviruses can reactivate and initiate a productive infection in the TG. New progeny virions can spread back to the primary replication site (36–38).

Although the general pathogenesis and common disease mechanisms of alphaherpesviruses are well described, little is known about the exact mechanism by which these viruses infect the URT epithelium and subsequently invade the lamina propria. Moreover, to date, it is generally accepted that primary replication of HSV-1 occurs at the mucocutaneous transition of the oral cavity, leading to the characteristic clinical manifestation of *herpes labialis* (39). Therefore, most research on HSV-1 has been performed on skin and oral tissues, and research into the URT as a possible primary site of replication has been neglected. Here, we performed a comparative study of the pathogenesis of three closely related, yet distinct alphaherpesviruses, within their natural hosts to identify possible commonalities as well as disparities between the different viruses at the level of their primary infection site. Discrepancies in replication kinetics in the epithelium, as well as efficiency of invasion of the BM and lamina propria, may explain the variability in clinical outcomes between species and be instrumental in pinpointing key steps in the early pathogenesis of alphaherpesviruses. At present, it remains unclear how herpesviruses succeed in crossing the BM and thereby distinguish themselves from other respiratory viruses, such as influenza viruses, which typically stay local in the respiratory tract (40). Here, we performed a comparative study of HSV-1, PRV, and BoHV-1 replication and invasion kinetics in the host URT to highlight key steps in their pathogenesis.

## MATERIALS AND METHODS

### Viruses

#### Wild-type (WT) viruses

All three wild-type alphaherpesvirus strains were available in the laboratory. PRV Becker strain was grown in swine testicle (ST) cells. BoHV-1 Cooper strain was grown in Madin-Darby bovine kidney (MDBK) cells. HSV-1 F strain was originally isolated from a human cold sore and grown in Afrikan green monkey kidney (VERO) cells. All cell lines used to propagate the viruses were kindly provided by Prof. Dr. M. Pensaert (Department of Virology, Immunology and Parasitology, Faculty of Veterinary Medicine, Ghent University). All viruses were used at their third passage. Inoculation was performed with a titer of 10^7.0^ TCID_50_/mL.

#### gE/gI null viruses

The gE/gI deletion mutants used in this study all arose from the parental wild-type strains mentioned above. The PRV Becker gE/gI null strain was already present in the laboratory. The BoHV-1 Cooper gE/gI null virus was designed in our laboratory using serial *en passant* mutagenesis (41). The gE/gI-null mutant of HSV-1 strain F (pGS7511) was produced from a self-excising bacterial artificial chromosome (BAC) infectious clone (pGS6000) that was previously modified to encode a pUL25/mCherry capsid tag (pGS6807) (REFS: PMC5006514, PMC6997764). The deletion spanned Us7 (encodes gI) and codons 1–443 of Us8 (encodes gE). A stop codon was placed before the remaining 109 codons of Us8, the latter of which lacked in-frame ATG codons. This residual Us8 sequence was retained to avoid disruption of downstream gene expression. Stocks of HSVF-GS7511 were produced by electroporation (BTX ECM 630 set to 950 µF, 200V, 0 Ω) of pGS7511 into Vero cells and harvested when all cells exhibited full cytopathic effect. The virus was serial passaged once on Vero cells (2 µL of harvested material was added to a confluent 10 cm plate of cells) to produce a high-titer stock. All mutant viruses were used at their third passage. Inoculation was performed with a titer of 10^7.0^ TCID_50_/mL.

### Collection and cultivation of respiratory explants for different species

#### Porcine respiratory mucosal explants

Six-week-old piglets were provided by the Flanders Research Institute for Agriculture, Fisheries and Food (ILVO). Upon arrival in the laboratory, the animals were euthanized using 1.25 mg/kg body weight natrium pentobarbital (Euthanimal, Kela, Belgium). The respiratory mucosa was stripped from the nasal septum using sterile surgical blades (Swann-Morton) and a tweezer. The mucosa was transferred into a Petri-dish containing Roswell Park Memorial Institute (RPMI) medium (ThermoFisher Scientific, Paisley, United Kingdom), supplemented with 100 U/mL penicillin, 0.1 mg/mL streptomycin, 0.1 mg/mL gentamicin, and 0.25 µg/mL amphotericin B (ThermoFisher Scientific). Square explants of 25 mm² were prepared and transferred onto sterile stainless steel grids (autoclavable), with a mesh-like structure and a pore size of 1 mm, allowing the culture medium to pass through and reach the bottom of the explants. Cultivation was done at an air-liquid interface for 24 h at 37°C and 5% CO_2_ in serum-free medium, containing 50% RPMI and 50% Dulbecco’s modified Eagle medium (DMEM) (ThermoFisher Scientific), supplemented with the above-mentioned antibiotics. In previous experiments, it was observed that the addition of fetal calf serum (FCS) to explant culture medium induces hyperplasia in epithelial cells, leading to a disruption of cell-cell contacts and a loss of their native three-dimensional structure (42). To preserve the integrity of the epithelial architecture and maintain physiologically relevant conditions in explant cultures, it has opted to use a serum-free medium.

#### Bovine respiratory mucosal explants

The proximal trachea was collected at the slaughterhouse from 6-month-old bovines. Upon collection, the tracheas were immediately submerged in 500 mL of transport medium consisting of phosphate-buffered saline (PBS) (ThermoFisher Scientific) with calcium and magnesium and supplemented with 100 U/mL penicillin, 0.1 mg/mL streptomycin, 0.1 mg/mL gentamicin, 0.1 mg/mL kanamycin (Merck, Darmstadt, Germany), and 0.25 µg/mL amphotericin B. In the laboratory, the respiratory mucosa was cautiously removed from the underlying cartilage and explants were cultivated in the same way as described above for the porcine tissues.

#### Human respiratory mucosal explants

Human nasal mucosa from the lower turbinate (concha nasalis inferior) was obtained at the University Hospital UZ Gent (Ghent, Belgium). The collection was performed at the time of surgery. Inferior turbinate removal surgery was performed for one of the following reasons: (i) septal deviation, (ii) nasal polyps, or (iii) rhinitis medicamentosa. Apart from those conditions, the patient’s nasal mucosa was healthy. Preparation and cultivation of the explants were performed using the same methods as described above.

### Opening of intercellular spaces by treatment with the calcium chelator ethylene glycol-bis (β-aminoethyl ether)-*N*,*N*,*N*′,*N*′-tetraacetic acid

This was performed as previously described, with minor modifications (28). Briefly, the explants were removed from the grids and placed in a 24-well plate after 18 h of cultivation. To remove the mucus layer, the explants were thoroughly washed with a serum-free medium. Treatments and subsequent inoculations were carried out using the agarose model as previously published (43). For the disruption of cell junctions, the apical side of the epithelium was exposed to 8 mM ethylene glycol-bis (β-aminoethyl ether)-*N*,*N*,*N*′,*N*′-tetraacetic acid (EGTA; Merck) in dPBS for 1 h at 37°C. Explants were submerged in 1 mL of serum-free medium or dPBS as a negative control. After treatment, the explants were washed three times in a serum-free medium. Afterward, the explants were either fixed in 3.6% formaldehyde for 24 h, in preparation for morphological analysis, or embedded in methylcellulose (methocel) and quick-frozen for viability studies, or the explants remained in the agarose for subsequent viral inoculation.

### Morphological assessment of the respiratory mucosa upon EGTA treatment

Immediately after the 1-h treatment with EGTA, the explants were washed and fixed in 3.6% formaldehyde for 24 h for morphological analysis. Paraffin embedding of the explants was carried out using an automated system (STP 420D, Micron, Praran, Merelbeke, Belgium). Consecutive sections of 8 µm thick were cut, deparaffinized in xylene, rehydrated in descending grades of alcohol, stained with hematoxylin and eosin (HE), dehydrated in ascending grades of alcohol and xylene, and mounted with DPX (DPX mountant, BDH Laboratory Supplies, Poole, UK). The percentage of intercellular space between the epithelial cells was evaluated by light microscopy and quantified using Image J software (Image J, U.S. National Institutes of Health, Bethesda, ML, USA). Briefly, a region of interest (ROI, i.e., the epithelium) was drawn manually in the “ROI manager tool.” Next, the threshold value to distinguish blank spaces from epithelial cells was determined, and the percentage of blank spaces between the cells (i.e., the intercellular space) was calculated, indicative of the junctional integrity.

### Inoculation of explants with wild-type and gE/gI deletion mutant viruses

After 1-h EGTA treatment, URT mucosal explants were inoculated with either WT or gE/gI null viruses. Inoculation was performed within the agarose model, as described above. Briefly, explants were washed three times in a warm serum-free medium. Next, explants were (mock) inoculated with the WT or gE/gI null viruses, diluted in 1 mL serum-free medium, supplemented with 1% penicillin and streptomycin and 0.5% gentamicin, to obtain a viral titer of 10^7.0^ TCID_50_/mL. Explants were incubated for 1 h at 37°C and 5% CO_2_. Explants were washed three times to remove unbound viral particles and placed back onto their grids, epithelium upwards. Cultivation was performed in an air-liquid interface in a serum-free medium supplemented with antibiotics. For the WT comparative analysis, explants from six biological replicates (independent animals) were pre-treated with EGTA and infected with WT strains of PRV, BOHV-1, and HSV-1. In two of these replicates, samples were fixed exclusively at 48 h post-infection (hpi) to assess viral infection under EGTA-treated conditions. For the third replicate, in addition to the 48 hpi fixation, a viral kinetics study was performed. Explants from this replicate were fixed at four distinct time points—12, 24, 48, and 72 hpi—to evaluate the temporal progression of viral infection. This third replicate, therefore, served as both the final biological replicate for the EGTA-treated condition and the first biological replicate for the viral kinetics analysis. To complete the viral kinetics study, fourth and fifth replicates were included, where explants were fixed at 12, 24, 48, and 72 hpi.

### Cryosectioning, immunofluorescent staining, and confocal microscopy

Viral- and mock-infected explants were frozen in methocel and stored at −70°C. In all, 20 consecutive sections of 12 µm were cut per explant, fixed with PFA (4%, 15 min, 4°C), and permeabilized (0.1% Triton X-100,10 min, RT). Viral replication was detected using mouse monoclonal anti-gB antibodies against PRV (1/100; IgG2a) (produced in our laboratory) BoHV-1 (1/100; IgG2b) (gift from Dr. van Drunen Littel), or HSV-1 (1/100; IgG2b) (ThermoFisher Scientific), followed by incubation with goat-anti-mouse IgG2a or IgG2b Alexa Fluor 488 (1/200; ThermoFisher Scientific) (44, 45). The BM and basal cells were stained using a mouse monoclonal IgG1 antibody against collagen type VII (1/300; Merck) and mouse monoclonal IgG2a antibody against cytokeratin 15 (CK15) (1/100; ThermoFisher Scientific), followed by goat-anti-mouse IgG1-Alexa Fluor 594 or IgG2a-Alexa Fluor 594 secondary antibodies (1/200; ThermoFisher Scientific). The integrin α6 was stained using a rat polyclonal antibody (1/50; ThermoFisher Scientific), followed by a goat-anti-rat-IgG FITC secondary antibody (1/100; ThermoFisher Scientific). Nuclei were counterstained with Hoechst 33342 (1/100; ThermoFisher Scientific). Slides were mounted with glycerol-DABCO and analyzed with a confocal microscope (Leica TCS SP2 Laser Scanning Spectral Confocal System; Leica Microsystems). The total number of plaques was counted in 20 cryosections (i.e., 2 mm^2^ epithelium) and the plaque diameter was measured using the Image J line tool. The percentage of infection was determined in a region of interest (ROI, i.e., epithelium), drawn manually for each picture in the “ROI manager tool.” The maximum invasion depth was measured with the Image J line tool. Information on the calculation of the percentage of the glycoprotein expression at the BM and the percentage of basal cells underneath plaque areas is given in the supplementary data (Materials and Methods and Fig. S1).

### Transmission electron microscopy of the epithelium and BM

Tissue samples were processed as previously described (46). Briefly, the explants were incubated overnight at 4°C in Karnovsky’s fixative [2% paraformaldehyde and 2.5% glutaraldehyde in 0.2 M sodium cacodylate buffer (pH 7.4)] (47). The samples were rinsed in 0.1 M sodium cacodylate buffer (pH 7.4), after which they underwent an overnight post-fixation procedure in 2% osmium tetroxide at 4°C. Each of the previous steps involved rotary motion of the solution during the incubation period. Dehydration was performed through a series of graded alcohols. The dehydrated samples were infiltrated with a low viscosity embedding (LVR) medium (Agar Scientific, Stansted, UK) for 2 days and then embedded in LVR. Ultrathin sections were cut using a glass knife on an Ultramicrotome Ultracut S (Leica, Vienna, Austria). Sections were stained with a Leica EM stain and examined on a JEM-1010 transmission electron microscope (Jeol Ltd., Tokyo, Japan) operating at 60kV.

### Inhibition of protease activity in porcine, bovine and human respiratory explants

The inhibition of protease activity in explants was performed as previously published by Glorieux et al., with minor adjustments (48). Briefly, prior to inhibition, explants were treated with 8 mM EGTA for 1 h, washed, and inoculated with WT virus. At 1 h post-inoculation, medium was replaced by medium supplemented with inhibitor for inhibitor-treated explants, or medium without inhibitor for mock-treated explants. Explants were immersed for 1 h and then transferred back to their respective gauzes and cultivated with a medium in the presence or absence of inhibitor until fixation. Fixation was performed at 36 hpi, as all three alphaherpesviruses were shown to invade the BM by that time.

In the first inhibitor experiment, a broad-acting protease inhibitor was used: the Complete Mini Protease Inhibitor Cocktail Tablets, containing a mixture of several protease inhibitors with broad inhibitory specificity for serine, cysteine, and metalloproteases (Roche Diagnostics Corporation, Basel, Switzerland). Inhibitor concentrations were used as recommended by the manufacturer’s instruction (one tablet complete Mini protease inhibitor cocktail per 10 mL). In a second experiment, the involvement of different protease families was investigated using family-specific protease inhibitors: pepstatin A (1 µg/mL) (Sigma, St. Louis, MO, USA) to inhibit aspartic proteases; AEBSF (4-(2-Aminoethyl) benzenesulfonyl fluoride hydrochloride) (250 µM) to inhibit serine proteases, E-64 (trans-Epoxysuccinyl-l-leucylamido-(4-guanidino)butane) (10 µM) to inhibit cysteine proteases and phosphoramidon (10 µM) (Sigma) to inhibit matrix metalloproteases. Tranexamic acid (TXA) (125 mM) (Sigma) was used to specifically block the trypsin-like serine protease urokinase plasminogen activator (uPA). Inhibitors that demonstrated a significant reduction in viral invasion were tested in three separate experiments, each using tissue from a different animal. Conversely, protease inhibitors that did not show a reduction in invasion depth were only tested in a single replicate. To analyze these experiments, 20 consecutive cryosections were prepared from each explant. The invasion depth (maximal plaque depth underneath the BM) was then measured for 10 distinct plaques within each explant, using the image analysis software Image J (Fiji).

### Statistical analysis

Data representation and statistical analysis were performed using GraphPad Prism 10 software. Statistical analysis was performed by two-way analysis of variance (ANOVA) followed by a multiple-comparison test (Tukey or Fisher’s LSD test). A value of *P*  <  0.05 was considered statistically significant. *P* values are depicted following the GraphPad Prism (GP) style: 0.1234 (ns), 0.0332 (*), 0.0021 (**), 0.0002 (***), and <0.0001 (****).

## RESULTS

### Removal of extracellular calcium enhances alphaherpesvirus infection in the host URT

**Fig 1:**
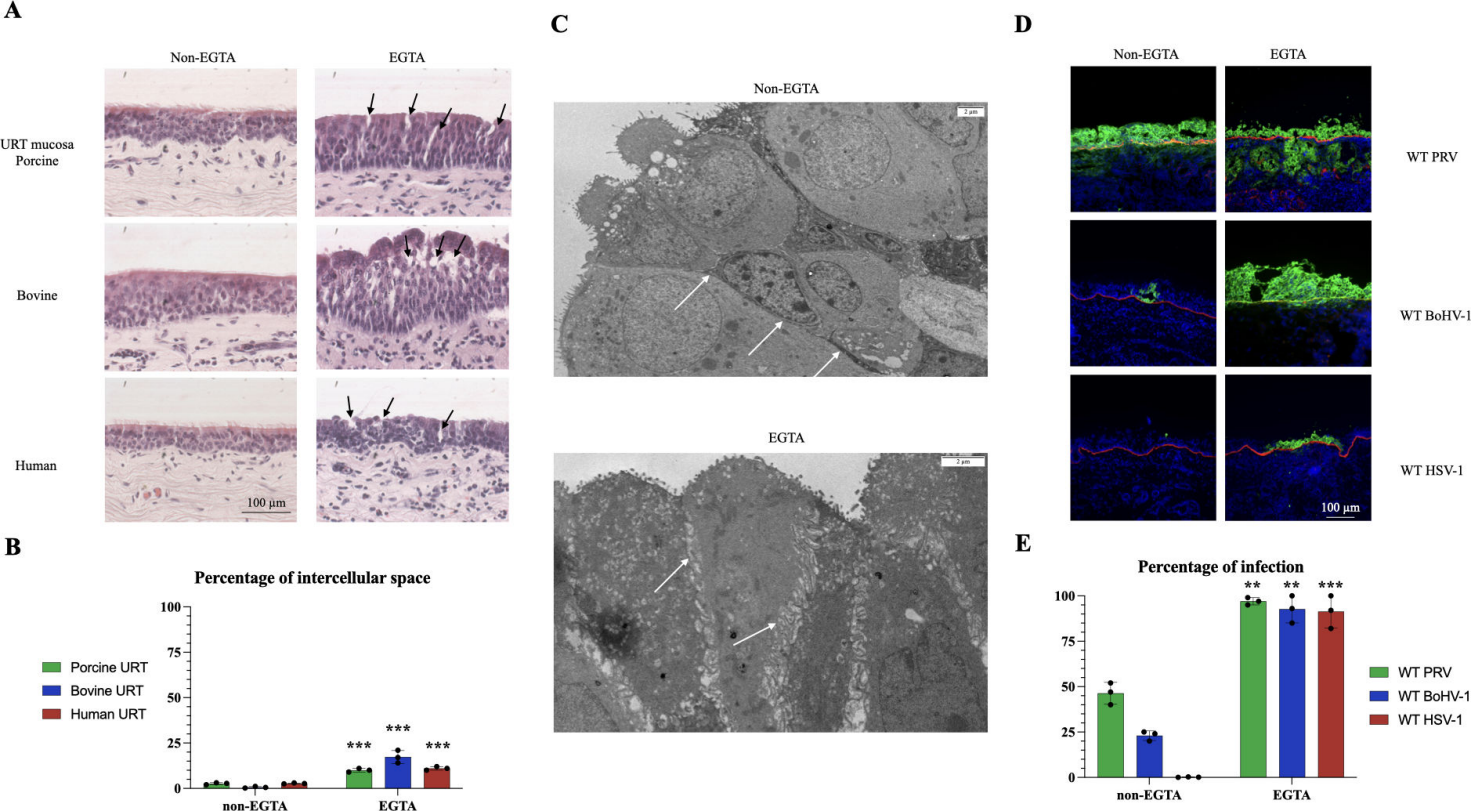
EGTA treatment of the respiratory mucosa destroys epithelial junctions and thereby enhances alphaherpesvirus infection in swine, bovine, and human explants. (**A**) Representative HE stainings showing the respiratory mucosa under normal conditions (left), and EGTA treatment (right) for the three different species. Arrows point out intercellular spaces. (**B**) Bar plot indicating central tendencies (mean ± SD) for the percentage of intercellular spaces in the epithelium. (**C**) Representative transmission electron microscopy pictures of non-EGTA (top) and EGTA-treated (bottom) porcine respiratory epithelium. White arrows point to cellular junctions. Scale bars represent 2 µm. (**D**) Representative immunofluorescent images of WT PRV, WT BoHV-1, and WT HSV-1 at 48 hpi, after serum-free medium treatment (negative control) (left column), or EGTA treatment (right column). The basement membrane (collagen type VII) is depicted by the red fluorescent color. (**E**) Bar plot depicting the percentage of WT PRV, WT BoHV-1, and WT HSV-1 infection in the epithelium at 48 hpi, after non-EGTA or EGTA treatment. Significant differences between the means of three independent experiments are depicted on the bar plots by asterisks (*P-value <0.05, **P-value <0.01, ***P-value <0.001, ****P-value <0.000.

We first evaluated whether the removal of extracellular calcium from the host URT mucosa with EGTA treatment could enhance HSV-1, PRV, and BoHV-1 infection. Damage to cellular junctions was evaluated by quantifying the percentage of intercellular spaces within the epithelium in HE sections. A significantly higher percentage of intercellular space was observed in the URT epithelium of all three species in EGTA-treated conditions compared to control (*P*-value < 0.001) (Fig. 1A through C). Second, we investigated whether the increase in intercellular spaces could subsequently enhance alphaherpesvirus infection. The percentage of infection in the URT epithelium significantly increased at 48 hpi from 0.1% ± 0.05 to 92.6% ±12.7 for HSV-1, from 47.3% ±8.2 to 97.3 % ±4.6 for PRV, and from 24.0% ±10.1 to 93.0 % ±6.5 for BoHV-1, following EGTA pretreatment (*P*-value < 0.01) (Fig. 1D and E). Therefore, we can conclude that the removal of extracellular calcium clearly disrupts the epithelial junctional integrity of the URT, leading to an enhanced viral infection in all three different species.

### WT PRV infects faster and invades deeper the URT of its host than WT BoHV-1 and HSV-1

**Fig 2:**
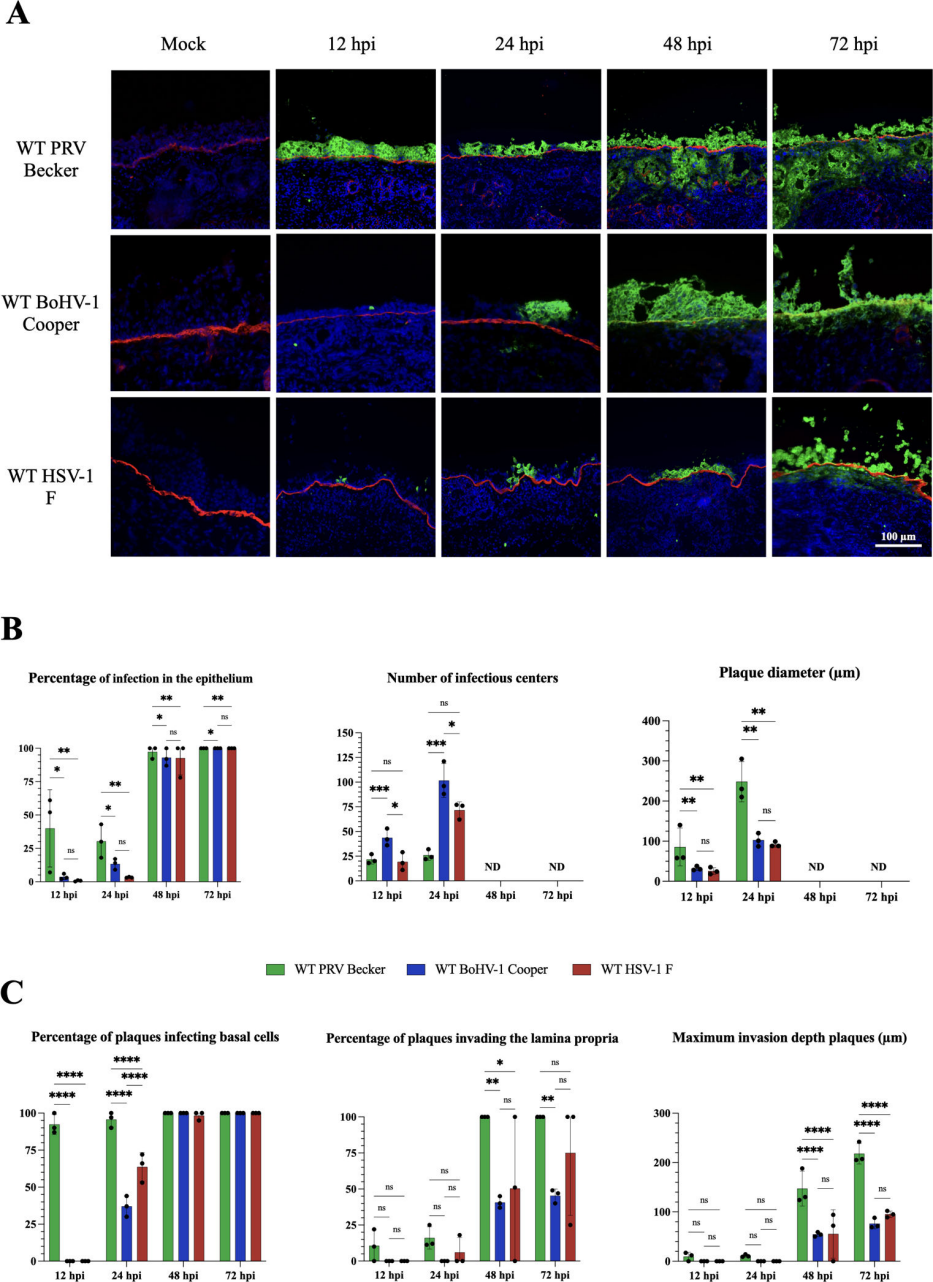
Infection and invasion of WT PRV, BoHV-1, and HSV-1 in porcine, bovine, and human respiratory mucosal explants. (**A**) Representative immunofluorescent pictures of uninfected URT (“mock”) and infected URT at 12, 24, 48, and 72 hpi. *Note*: The images used in Fig. 2A (48 hpi) are the same as those used in Fig. 1D (right column), as they represent identical experimental conditions (EGTA-treated explants inoculated with WT virus at 48 hpi) across both figures. This duplication reflects consistent observations from replicate experiments. The glycoprotein B (gB) of each alphaherpesvirus was stained in green (AlexaFluor 488) and the BM (collagen type VII) was stained in red (AlexaFluor 594). Nuclei were counterstained in blue (Hoechst 33342) (scale bar represents 100 µm). (**B**) Central tendencies for the amount of infection in the respiratory epithelium are indicated by the upper three bar plots [left graph: percentage of infection in the epithelium; middle graph: number of infectious centers (i.e., viral plaques, SIC and groups of 2–3 infected cells)/2 mm^2^ epithelium; right graph: average plaque diameter (µm)]. (**C**) Viral invasion parameters are presented in the three lower bar plots [left graph: percentage of plaques infecting basal cells; middle graph: percentage of plaques crossing the BM and invading the lamina propria; right graph: maximum invasion depth of the plaques underneath the BM (µm)]. Values in all graphs are presented as means ± standard deviations (SD). Significant differences between the means of three independent experiments are depicted on the bar plots by asterisks (*P-value <0.05, **P-value <0.01, ***P-value <0.001, ****P-value <0.0001).

Next, we compared the replication kinetics and invasion efficiency of the three distinct alphaherpesviruses in their host URT. Respiratory explants were infected with WT viruses, following EGTA treatment. Viral replication kinetics in the respiratory mucosa was evaluated by IF staining. At 12 and 24 hpi, we found that the percentage of PRV-infected epithelial cells was significantly higher than that of HSV-1-infected and BoHV1-infected epithelial cells (*P*-value < 0.05) [Fig. 2A and B (left side)]. The percentage of infection in the epithelium reached 100% in all three infections. We also counted the number of infectious centers that included single infected cells (SIC), groups of two to three infected cells, as well as viral plaques (i.e., more than three adjacent infected cells). As seen in Fig. 2B (middle graph), BoHV1-infected explants showed the highest number of infectious centers (101.6 ± 17.2) per 2 mm^2^ epithelium, followed by HSV-1- (71.6 ± 8.3) and PRV-infected explants (26.0 ± 5.2) at 24 hpi. The number of infectious centers could not be determined at 48 and 72 hpi due to the high level of infection. We measured the plaque diameter and showed that PRV plaques were significantly bigger (85.6 µm ± 47.0) than those of BoHV-1 or HSV-1 (32.0 µm ± 5.2 and 25.6 µm ± 8.6, respectively) [Fig. 2B (right graph)]. The plaque diameter increased for all three viruses at 24 hpi, showing PRV plaques twofold bigger than BoHV-1 and HSV-1 plaques. Plaque diameters were not determined and infectious centers could not be distinguished separately.

We then quantified the percentage of plaques that infected basal cells (i.e., a plaque with a green fluorescent signal in the epithelial cells directly adjacent to the BM). As shown in Fig. 2C (left graph), the percentage of PRV plaques infecting basal cells was 92.3% ± 6.8% while no basal cells were infected in BoHV-1 and HSV-1 conditions. At 24 hpi, the percentage of BoHV-1 and HSV-1 plaques infecting basal cells increased to 37.0% ± 7% and 63.6 % ± 9.7%, respectively. Starting from 48 hpi, all basal cells were infected, independent of the virus used. We also determined the percentage of plaques invading the lamina propria, which was defined as the number of plaques showing colocalization with the BM, as well as a fluorescent signal underneath the BM. As shown in Fig. 2C (middle graph), PRV invasion kinetics started as early as 12 hpi with 10.6% ± 11.0% of plaques crossing the BM while HSV-1 invasion was delayed until 24 hpi. BoHV-1 invasion was only observed starting from 48 hpi with 40.6% ± 4.0% of plaques invading the lamina propria. At 72 hpi, 100% of PRV plaques invaded the lamina propria while a lower percentage of invading plaques was observed during HSV-1 (75.0% ± 43.3%) and BoHV-1 (45.3% ± 4.7%) infections. Finally, we measured the maximum invasion depth of a plaque, which was defined as the longest distance measured toward the lamina propria, starting perpendicular to the BM. In case separate plaques could be distinguished, the average of 10 individual plaques was determined. When separate plaques could not be distinguished, the maximum invasion depth was measured at 10 randomly chosen spots within the infected area (at 48 and 72 hpi for all three viruses). As seen in Fig. 2C (right graph), only the maximum invasion depth of PRV plaques could be determined starting from 12 hpi (9.6 µm ± 8.7%). It increased over time and reached 218.0 µm ± 20.8 at 72 hpi. By contrast, the maximum invasion depth of HSV-1 and BoHV-1 plaques could only be measured at 48 hpi and slightly increase at 72 hpi (76.0 µm ± 10.4 for BoHV-1 and 95.3 µm ± 6.5 for HSV-1). Overall, these data clearly demonstrated that all three wild-type herpesviruses can infect the respiratory epithelium of their host and invade the lamina propria. However, we demonstrated that PRV infects more rapidly and more extensively the epithelium compared to HSV-1 and BoHV-1. We also showed that PRV invades faster the lamina propria, followed by HSV-1 and BoHV-1.

### WT PRV glycoproteins B and D differ in their polarized expression compared to WT HSV-1 and WT BoHV-1

To examine whether the expression kinetics and localization of envelope glycoproteins may explain the different invasion dynamics of PRV, BoHV-1, and HSV-1, we systematically assessed the expression profiles of the main envelope glycoproteins gB, gC, gD, and gE in the respiratory epithelial cells at the level of the BM. The method of calculating the amount of glycoprotein expression at the BM is given in the supplementary data (Fig. S1).

**Fig 3:**
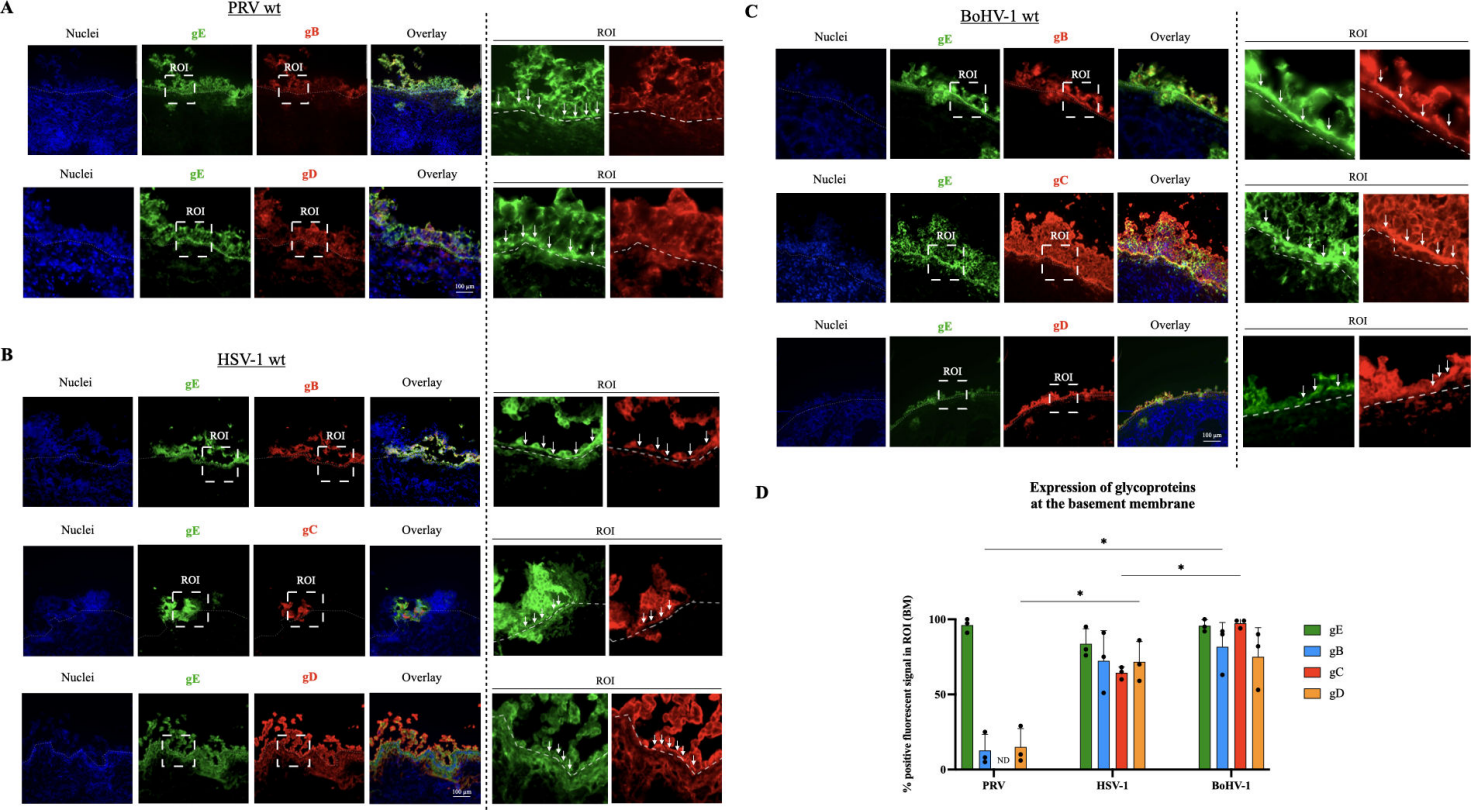
Polarized expression of glycoprotein B, C, D, and E of WT PRV, HSV-1, and BoHV-1 in the URT epithelium. (**A, B, and C**) Representative immunofluorescent pictures of WT PRV, WT HSV-1, and WT BoHV-1 infection and invasion at 48 hpi. The glycoprotein E (gE) of each alphaherpesvirus was stained in green (AlexaFluor 488) and the glycoproteins B, C, and D (gB, gC, and gD) were stained in red (AlexaFluor 594). Nuclei were counterstained in blue (Hoechst 33342). The BM is depicted by a white dashed line. The scale bar represents 100 µm. An ROI around the BM was enlarged to show the polarization. White arrows point out glycoprotein expression at the BM. (**D**) Central tendencies for the percentage of glycoprotein expression at the level of the BM are indicated by the bar plot. Values in the graph are presented as means ± standard deviations (SD). Significant differences between the means of three independent experiments are depicted on the bar plots by asterisks (*P-value <0.05).

At the moment of invasion (48 h post-infection), a notable proportion of gE expression was observed at the BM for all three viruses, with percentages as follows: 96.1% ± 4.%6 in WT PRV, 83.7% ± 10.0% in WT HSV-1, and 95.7% ± 4.0% in WT BoHV-1 (Fig. 3A through D). The expression levels of glycoproteins B, C, and D at the BM were also markedly elevated in WT HSV-1 and BoHV-1. HSV-1 gB expression was 72.3% ± 20.1%. This was similar for gD (71.6% ± 13.5%) and slightly lower for gC (64.3% ± 4.0%) (Fig. 3B and D). Expression levels of BoHV-1 gB, gC, and gD glycoproteins amounted to 97.3% ± 2.8% for gC, 81.6% ± 16.1% for gB, and 75.0% ± 19.4% for gD (Fig. 3C and D). By contrast, the expression of WT PRV gB and gD at the BM (gC expression could not be determined due to the lack of antibodies) was markedly lower, with values of 12.6% ± 10.7% and 15.0% ± 12.2%, respectively (Fig. 3A and D).

### Protease-dependent invasion in WT HSV-1 and uPA-mediated WT PRV invasion, contrasted with WT BoHV-1′s protease-independent mechanism

**Fig 4:**
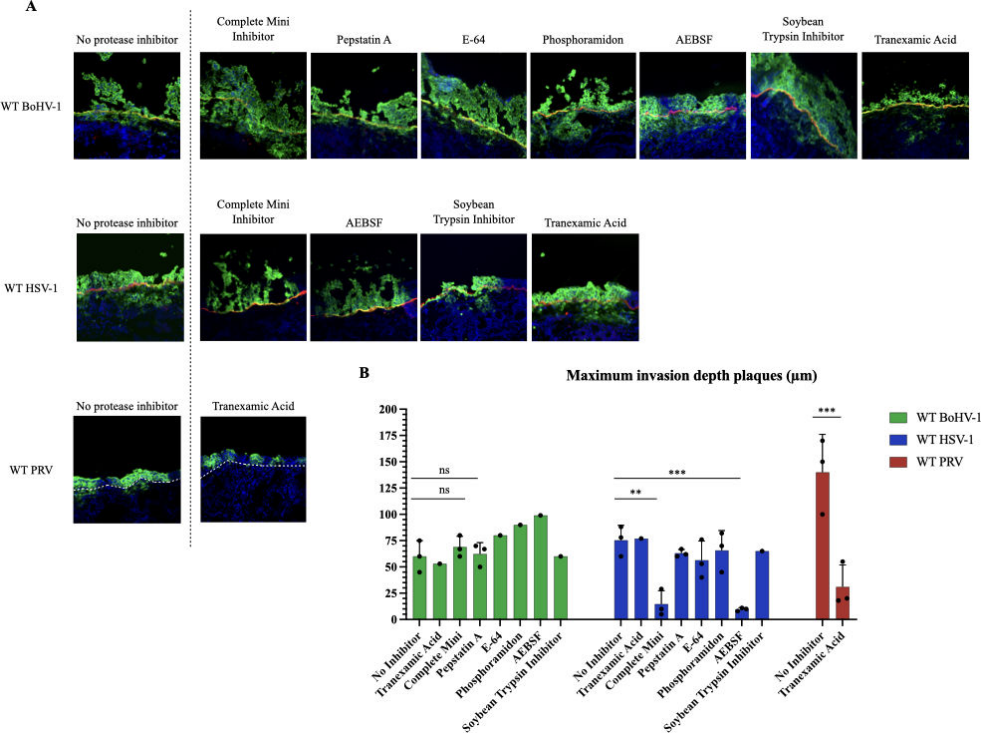
Inhibition of serine proteases in WT PRV and WT HSV-1 reduces BM invasion, contrary to WT BoHV-1. (**A**) Representative immunofluorescent pictures at 36 hpi. The glycoprotein B (gB) of each alphaherpesvirus was stained in green (AlexaFluor 488) and the BM (collagen type VII) was stained in red (AlexaFluor 594). Nuclei were counterstained in blue (Hoechst 33342) (pictures taken with a 20x objective, scale bar represents 100 µm) (**B**) The bar plot depicts the maximum invasion depth underneath the BM (µm) at 36 hpi, after protease inhibitor or control treatment. Each black dot represents one replicate value (=1 animal experiment). The colored bars represent the mean values. Significant differences between the means of three independent experiments are depicted on the bar plots by asterisks (*P-value <0.05, **P-value <0.01, ***P-value <0.001).

In the past, our laboratory identified a trypsin-like serine protease as being important for the invasion of PRV through the BM and underlying connective tissues. Nothing was known about the importance of proteases in the spread of BoHV-1 and HSV-1. Consequently, we subjected explants to treatment with protease inhibitors. In addition, we tried to identify the specific protease responsible for mediating WT PRV invasion. The results are depicted in Fig. 4A and B. Without inhibitor treatment, BoHV-1 reached a maximum invasion depth of 60.5 µm ± 15.0. After treatment with the different protease inhibitors, this invasion depth remained similar, ranging from 53.3 µm to 80.5 µm, or even increased, in the case of treatment with phosphoramidon (90.0 µm) and AEBSF (99.1 µm). By contrast, HSV-1 invasion depth decreased significantly after treatment with the Complete Mini cocktail (14.6 µm ± 12.6) and AEBSF (9.8 µm ± 1.5), compared to the negative control (75.5 µm ± 14.2). Treatment with Pepstatin A, E-64, phosphoramidon, soybean trypsin inhibitor, and tranexamic acid had no significant effect on the invasion depth of HSV-1.

For WT PRV, it was previously demonstrated that the soybean trypsin inhibitor blocked viral invasion underneath the BM, indicating the involvement of a trypsin-like serine protease. Here, we used tranexamic acid (TXA), a specific inhibitor of the trypsin-like serine protease urokinase plasminogen activator (uPA). TXA treatment resulted in a marked decrease in invasion depth, compared to the negative control (from 140.5 µm ± 36.5 to 31.4 µm ± 20.7).

Based on the results, we may conclude that uPA is facilitating the spread of PRV through BM and underlying connective tissues. The spread of HSV-1 is also helped by a serine protease, different from uPA. Proteases are not involved in the spread of BoHV-1 in the respiratory mucosa.

### The gE/gI glycoprotein complex is required for efficient alphaherpesvirus invasion of the URT mucosa

Considering the results above, it was concluded that proteases contribute to the invasion process of WT PRV and WT HSV-1, but not WT BoHV-1. However, the incomplete hindrance of BM invasion suggests the involvement of additional mechanisms. Among the potential viral factors, the glycoprotein complex gE/gI assumes significance, as it is known to have a role in facilitating cell-to-cell spread, thereby implicating it in the invasion process (49–51). To investigate the role of gE/gI, URT mucosal explants were infected with PRV, HSV-1 or BoHV-1 gE/gI null mutant viruses.

**Fig 5:**
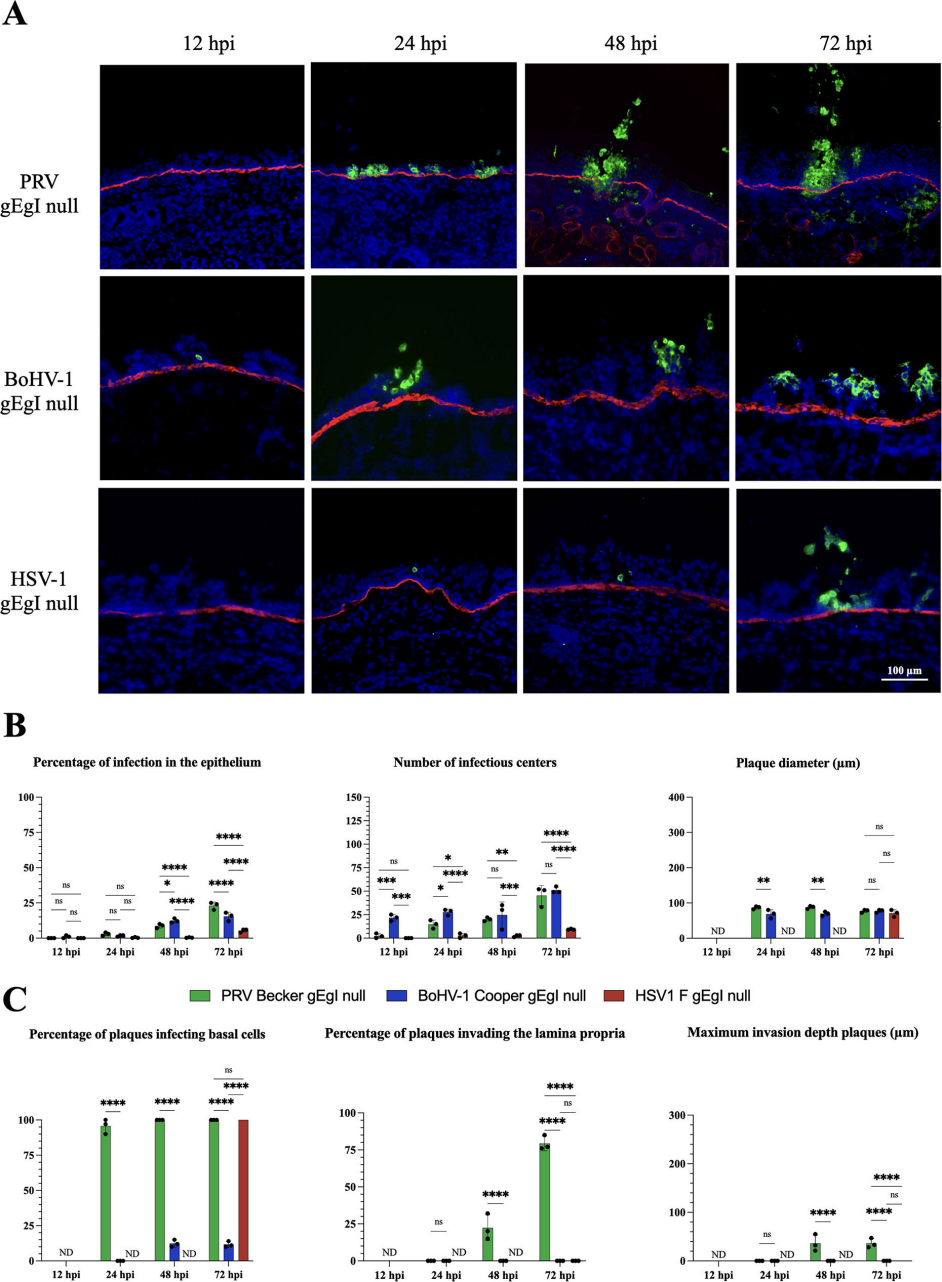
Comparative replication kinetics of PRV, BoHV-1, and HSV-1 gE/gI deletion mutants (gE/gI null). (**A**) Representative immunofluorescent pictures at 12, 24, 48, and 72 hpi. The glycoprotein C (gC) of each alphaherpesvirus was stained in green (AlexaFluor 488) and the BM (collagen type VII) was stained in red (AlexaFluor 594). Nuclei were counterstained in blue (Hoechst 33342) (pictures taken with a 20x objective, scale bar represents 100 µm). (**B**) Central tendencies for the amount of infection in the respiratory epithelium are indicated by the upper three bar plots [left graph: percentage of infection in the epithelium; middle graph: number of infectious centers (i.e. viral plaques, SIC and groups of 2–3 infected cells)/2 mm^2^ epithelium; right graph: average plaque diameter (µm)]. (**C**) Viral invasion parameters are presented in the three lower bar plots [left graph: percentage of plaques infecting basal cells; middle graph: percentage of plaques crossing the BM and invading the lamina propria; right graph: maximum invasion depth of the plaques underneath the BM (µm)]. Values in all graphs are presented as means ± standard deviations (SD). Significant differences between the means of three independent experiments are depicted on the bar plots by asterisks (*P-value <0.05, **P-value <0.01, ***P-value <0.001, ****P-value <0.0001).

As shown in Fig. 5A and B (left graph), PRV and BoHV-1 gE/gI null mutants started to infect the respiratory epithelium at 12 hpi while HSV-1 gE/gI-infected epithelial cells were only detected at 24 hpi. The percentage of infection increased throughout the course of the experiment and reached 23.0% ± 2.6%, 15.3% ± 3.0%, and 5.3 % ± 1.1% at 72 hpi for PRV, BoHV-1, and HSV-1 infection, respectively. As shown in Fig. 5B (middle graph), the number of infectious centers for PRV gE/gI null infection increased from 2.3 ± 2.5 at 12 hpi per 2 mm^2^ epithelium to 20.0 ± 2.0 at 48 hpi while those for BoHV-1 infection remained steady (24.6 ± 13.7). At 72 hpi, both PRV gE/gI null and BoHV-1 gE/gI null showed a comparable number of infectious centers (45.3 ± 10.6 and 51.0 ± 3.4, respectively). HSV-1 infectious centers were only detected starting from 24hpi (2.6 ± 2.5) and the number increased to 9.3 ± 0.5 at 72 hpi. The plaque diameter for PRV gE/gI null and BoHV-1 gE/gI null mutants could only be determined starting from 24 hpi because infectious centers only appeared as SIC or as groups of two adjacent infected cells with both viruses at 12 hpi. The plaque diameter was comparable for both viruses and remained similar over time (approx. 86.6 µm ± 4.0). The plaque diameter for HSV1 gE/gI null mutant was only determined at 72 hpi and was comparable to PRV gE/gI null and BoHV-1 gE/gI null mutants.

As seen in Fig. 5C (left graph), approximately 90% of PRV gE/gI null plaques infected basal cells starting from 24 hpi and reaching 100% at 72 hpi. A high percentage of HSV1 gE/gI null plaques infecting basal cells (100.0%) was only detected at 72 hpi. By contrast, BoHV-1 gE/gI null did not infect basal cells until 48 hpi, where a significantly lower percentage of plaques infecting basal cells (12.3% ± 2.5%) was quantified compared to PRV gE/gI null. This percentage remained similar at 72 hpi. No plaques invaded the lamina propria for both BoHV-1 gE/gI null and HSV-1 gE/gI null viruses throughout the course of the experiment [Fig. 5C (middle graph)]. By contrast, PRV gE/gI null did invade the lamina propria, however only starting from 48 hpi (22.3% ± 8.7%). The percentage of invasion increased to 79.3% ± 4.9% at 72 hpi. Consequently, the maximum invasion depth could not be determined for BoHV-1 gE/gI null and HSV-1 gE/gI null mutants. For PRV gE/gI, the maximum invasion depth was 36.0 µm ± 17.3 at 48 hpi and remained similar until 72 hpi (36.0 µm ± 9.8) [Fig. 5C (right graph)]. Here, we clearly demonstrated that the alphaherpesvirus gE/gI glycoprotein complex is essential for efficient infection of the host respiratory epithelium and invasion through the BM. Viral infection and invasion of the URT were significantly reduced and delayed with gE/gI null mutants compared to WT strains (Fig. 2). Differences were also observed among the three gE/gI deletion mutant strains. While PRV gE/gI null and HSV-1 gE/gI null were hampered in their invasion through the BM, the BoHV-1 gE/gI null mutant was fully blocked. The latter even failed to infect the basal cell layer of the epithelium.

### Proliferating basal cells are resistant to BoHV-1 gE/gI null infection

**Fig 6:**
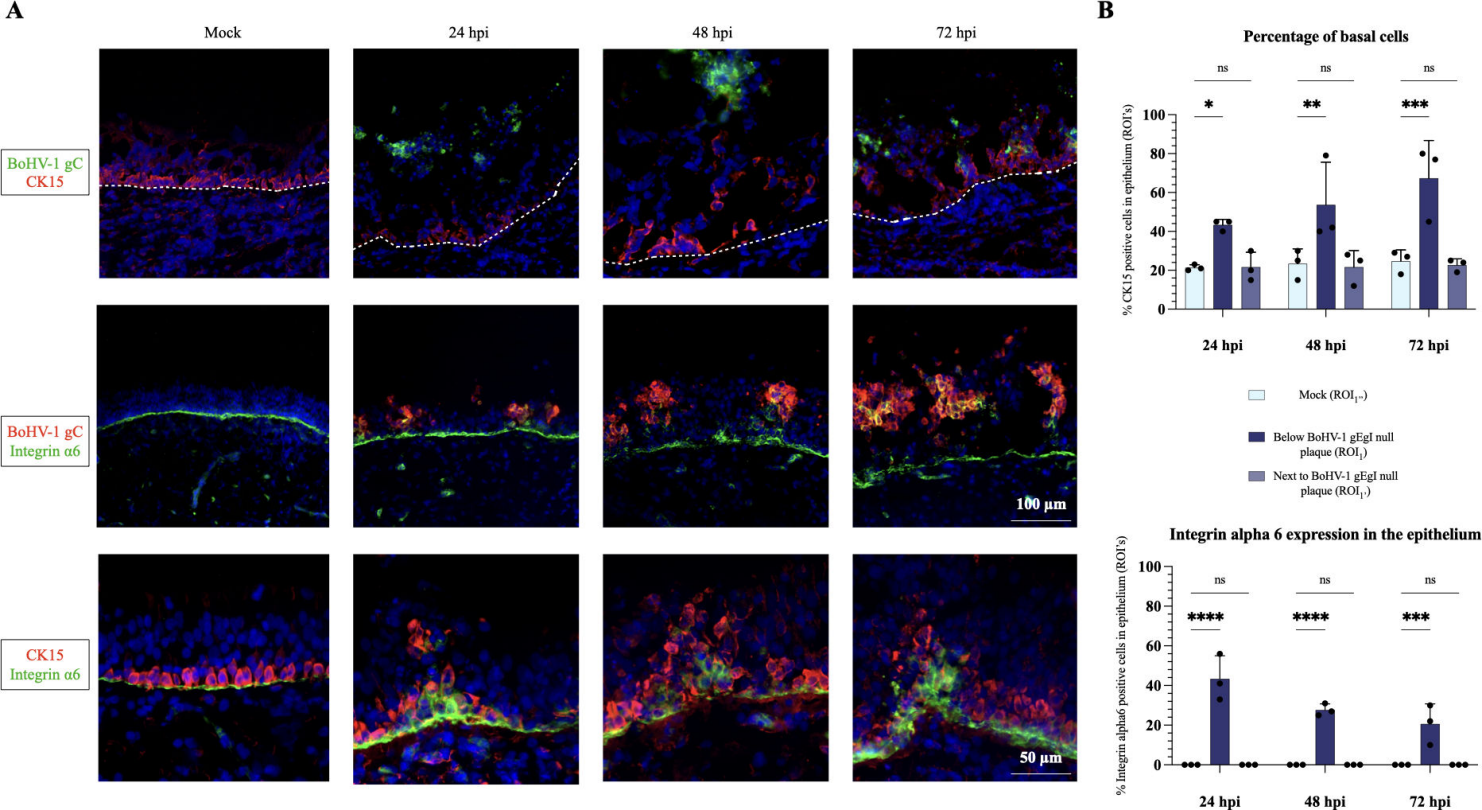
Proliferating basal cells are resistant to BoHV-1 gE/gI null infection. (**A**) Representative immunofluorescent pictures. *Top row*: the glycoprotein C (gC) of BoHV-1 gE/gI null is stained in green (AlexaFluor 488), the basal cells (CK15) are stained in red (AlexaFluor 594). The dashed line represents the BM. *Middle row:* the glycoprotein C (gC) is stained in red and the integrin α6 is stained in green. *Bottom row:* The basal cells are shown in red and the integrin α6 is shown in green. Nuclei were counterstained with Hoechst. The scale bar of the top and middle row represents 100 µm. The scale bar of the bottom row represents 50 µm. (**B**) *Top graph:* the percentage of basal cells in the epithelium. Central tendencies for the percentage of CK15-positive cells are depicted in different ROIs. (ROI_1_ = area underneath a viral plaque in BoHV-1 gE/gI null infected epithelium; ROI_1’_ = an area in the infected epithelium, adjacent to a viral plaque; ROI_1’’_ = an area of similar size in mock-inoculated epithelium). *Bottom graph:* The expression of integrin α6 in the epithelium. Central tendencies for the percentage of integrin α6-positive cells are depicted in the same ROI’s as mentioned above. Values in all graphs are presented as means + standard deviations (SD). Significant differences between the means of three independent experiments are depicted on the bar plots by asterisks (*P-value <0.05, **P-value <0.01, ***P-value <0.001, ****P-value <0.0001).

Intriguingly, the BoHV-1 gE/gI complex seems critical for both invasion of the BM and infection of basal cells. To confirm that BoHV-1 gE/gI null specifically does not infect basal cells, we performed a double IF staining for viral antigens (gC) and cytokeratin 15 (CK15) (basal cell marker). In mock-inoculated explants, basal cells were located as a single-cell layer present at the bottom of the epithelium, directly adjacent to the BM [Fig. 6A (top row)]. In BoHV-1 gE/gI null-infected explants, we showed that basal cells indeed remained uninfected until 72 hpi. Intriguingly, we found that the number of basal cells underneath infected cells increased compared to mock-inoculated explants. Approximately 67% of cells showed a CK15 positive signal underneath viral plaques (ROI_1_) at 72 hpi, compared to 20% of basal cells in mock explants (ROI_1’’_). As shown in Fig. 6A (top and bottom rows), basal cells appeared in the form of a triangle underneath the viral plaques. To validate this, we performed an additional IF staining with another basal cell marker (integrin α6 subunit). In mock-inoculated explants, we found that the integrin α6 was expressed at the basolateral side of basal cells, appearing as a thin line at the level of the BM [Fig. 6A (middle and bottom row)]. No integrin α6 expression was observed inside the epithelium. By contrast, BoHV-1 gE/gI null-infected explants showed an upregulation of integrin α6 expression underneath viral plaques, ranging from 40% at 24 hpi, to 20% at 72 hpi compared to mock [Fig. 6B (bottom graph)]. Figure 6A (bottom row) shows a clear colocalization of the CK15 and integrin α6 signals underneath BoHV-1 gE/gI null plaque areas (ROI_1_). These results clearly demonstrate that basal cells are not only resistant to BoHV-1 gE/gI null infection but respond to infection by cell proliferation and by downregulating the expression of integrin α6 protein.

## DISCUSSION

Alphaherpesviruses are clinically and economically important pathogens in mammals worldwide. So far, the molecular mechanisms underlying their replication and invasion of the host URT are not known. Here, we performed a comparative pathogenesis study of HSV-1, PRV, and BoHV-1 infections using well-established, fully standardized, species-specific *in-house* mucosal explants. First, we showed that all three alphaherpesviruses could infect the URT epithelium of their host with PRV being the most efficient one. Interestingly, we also demonstrated that the removal of extracellular calcium from the explant environment significantly enhances all three infections by disrupting calcium-dependent cellular junctions in the epithelium. These findings are in line with a previous study, which demonstrated that EHV1 infection is enhanced in the equine respiratory mucosa following EGTA treatment (28). The authors also showed that the enhanced susceptibility was due to EHV-1 targeting a basolateral receptor in the URT epithelium. Altogether, these findings suggest that the use of a basolateral receptor is not a unique strategy of EHV-1 to invade the URT, but rather a common invasion mechanism shared by several alphaherpesviruses. Furthermore, these results imply that environmental hazards that disrupt cellular junctions may increase the susceptibility of the host’s URT to viral infections. Indeed, multiple environmental factors have already been shown to enhance EHV-1 infection in the equine respiratory mucosa. These factors include mycotoxin deoxynivalenol, several pollen proteases, certain bacterial toxins, and proteases from *Aspergillus fumigatus* (47, 49, 50, 52). It is probable that other conditions damaging the respiratory mucosa, such as allergies, may also lead to increased infection and invasion of herpesviruses in the respiratory mucosa.

Second, we showed that all three herpesviruses replicated in the URT starting from 12 hpi. However, PRV was the earliest and most efficient virus to replicate in the epithelium and to invade the lamina propria compared to BoHV-1 and HSV-1. Interestingly, plaque formation was seen as early as 12 hpi in WT PRV. These findings can directly be correlated to differences observed in the severity of clinical symptoms between species. Indeed, respiratory infection with PRV produces considerably harsher symptoms in pigs (high fever and a necrotizing mucosa) than in cattle (mainly light respiratory symptoms, such as nasal discharge and a cough) (51, 53). Interestingly, we observed that the bovine respiratory epithelium is markedly thicker compared to porcine and human mucosa, which might contribute to the observed differences in susceptibility to alphaherpesvirus infection. We hypothesized that the impaired infection and invasion observed with BoHV-1, as compared to PRV, may be partially attributed to an intrinsic species-specific resistance present in the bovine epithelium. To explore this hypothesis, we conducted an experiment in which bovine respiratory epithelium was exposed to PRV, which has a broad host range. Despite PRV’s known ability to infect bovines, we were unable to establish infection in the bovine respiratory epithelium, even with the use of EGTA to compromise cellular junctions. This suggests that the structural and physiological characteristics of the bovine epithelium may render it more resistant to viral infection and invasion. Furthermore, the more restricted HSV1 replication and invasion, compared to PRV, might explain why humans rarely experience respiratory symptoms following infection (54). It is believed that the virus tends to preferentially infect the mucocutaneous surface of the oral cavity for efficient replication (54). However, in the present study, we demonstrated that the human URT is also a primary HSV1 replication site that should get more attention, especially in multifactorial diseases. An adaptation of the diagnostics is essential in this context. In the veterinary world, co-infections have been demonstrated to be involved with respiratory complexes and therefore, new diagnostic platforms have been launched, based on third-generation sequencing. One of these platforms, PathoSense is already commercially active. It is advisable to introduce this technology for routine analyses in human medicine.

To investigate whether variations in the invasion dynamics of PRV, BoHV-1, and HSV-1 could be attributed to the differential expression of key envelope glycoproteins gB, gC, gD, and gE, we analyzed these glycoproteins’ expression patterns in the respiratory mucosa. First, we showed that the glycoprotein E is expressed in a polarized manner in all alphaherpesviruses. High expression of gE is detected at the level of the BM. A polarized, lateral expression of HSV-1 gE was demonstrated before in HEC-1A cells (55). Second, we demonstrated that WT HSV-1 and BoHV-1 glycoproteins B, C, and D are similarly highly expressed at the level of the BM. This is in contrast with WT PRV, where glycoproteins B and D do not exhibit a polarized expression at the BM. It was clear that a high expression of gE together with gB, gC, and gD at the BM was associated with a rather slow invasion. By contrast, expression of gE alone at the BM, as seen in WT PRV, was strongly facilitating spread through this barrier. These results demonstrate that gE, when acting alone, enhances penetration. Why gE alone is that important in invasion, was enigmatic at that stage of our experiments.

In a previous study from our laboratory, we showed that a trypsin-like serine protease plays a crucial role for PRV in penetrating the BM and underlying connective tissues. The plaque invasion depths were reduced by 90% using a Soybean trypsin inhibitor in porcine respiratory explants (48). The involvement of protease activity, likely breaking down the structure of the BM, could explain the more rapid and unhindered invasion of WT PRV, compared to that of WT HSV-1 and WT BoHV-1. In this study, we continued to investigate the protease involvement in the invasion of the three alphaherpesviruses. For WT BoHV-1, protease activity was not important in the BM crossing. HSV-1 invasion was helped by a serine protease, but not by trypsin-like serine proteases, like for PRV. In addition, in the current study, we were able to identify the trypsin-like serine protease active in WT PRV invasion as the urokinase plasminogen activator (uPA), using its specific inhibitor tranexamic acid (TXA). In addition, we attempted to perform immunofluorescent staining for uPA in PRV-infected explants at different hpi (20, 24, 36, and 48). However, we could not detect uPA in the epithelium. As a control for the efficacy of the antibody used, we successfully stained HEK-T cells transfected with PLAU cDNA, coding for uPA. More details are given in the supplementary data (Materials and Methods and Fig. S3).

Integrating our findings on the polarization of glycoproteins with protease activity, we have formulated the following hypothesis: glycoprotein E exhibits a consistent polarized expression at the basal side of epithelial cells, near the BM. This positioning underlines the crucial role of the gE in the invasion process. It is well established that the gE/gI glycoprotein complex is expressed in a polarized manner in infected cells. Within neuronal systems, this complex engages the US9 viral protein, which, in turn, specifically associates with the kinesin motor protein KIF1A, facilitating anterograde movement (56). We propose that a similar mechanism operates in respiratory epithelial cells, wherein gE binds to a kinesin motor protein oriented exclusively toward the cell’s basal side. Subsequently, the extracellular domain of gE interacts with specific proteins, directing them toward the basal region. In the context of neurons, gE mediates the anterograde transport of virions (57). Analogously, we posit that in respiratory epithelial cells, gE might either bind principal glycoproteins involved in fusion and binding—such as gB, gC, and gD, similar to the mechanisms observed in BoHV-1 and, to a lesser extent, HSV-1—or engage proteases essential for degrading the BM to facilitate invasion, as seen in PRV and, again, to a lesser extent in HSV-1. The observed discrepancies in glycoprotein versus protease binding preferences may be attributed to distinct differences in the tertiary structure of the extracellular domain of gE across the three viruses, potentially explained by viral evolution. This hypothesis is however difficult to assess since X-ray crystallographic analysis of the tertiary structure thus far have only been performed for HSV-1 F, and not for PRV Becker or BoHV-1 Cooper. In the past, before the intensification of animal husbandry and before the explosive growth of the human population, all alphaherpesviruses most probably exhibited a main genital tract tropism. Over time, the increase in the density of animals and humans allowed them to adapt to the respiratory tract, presumably due to the enhanced efficiency of respiratory transmission. Also, the use of artificial insemination in swine and cattle effectively blocked the spread through the genital route. PRV underwent this tropic transition significantly earlier than HSV-1 and BoHV-1, potentially explaining its more efficient respiratory system adaptation, with a more effective epithelial barrier penetration and BM invasion. The preferential binding of gE to proteases rather than to glycoproteins may confer an evolutionary advantage, suggesting a potential future evolutionary trajectory for HSV-1 and BoHV-1 toward employing proteases for invasion. HSV-1 is already used in part proteases to cross the BM. This is a dangerous evolution and should be considered a threat to humans. Consequently, it is important to be prepared. An effective HSV-1 vaccine is urgently needed.

Building upon our findings regarding the polarized expression of viral glycoproteins and proteases by gE, our investigation delved into the specific examination of the role of gE in the behavior of the three examined alphaherpesviruses. Infection with gE/gI null viruses was delayed and reduced in the epithelium compared to WT infection. The percentage of infection and number of plaques was markedly lower and plaque diameters were significantly smaller for all infections. Surprisingly, the BoHV-1 gE/gI mutant initiates infection as early as 12 hpi, whereas PRV and HSV-1 mutants are only detected at 24 hpi. This discrepancy may be attributable to functional differences in the gE/gI complexes among these viruses. To explore this hypothesis, we conducted an alignment of the amino acid sequences, revealing significant differences in the gE proteins. Specifically, a 25.84% identity between the gE of PRV and BoHV-1 and a 33.47% identity between the gE of HSV-1 and BoHV-1 was found. Substantial differences in functionality might explain the observed discrepancies in mutant BoHV-1 versus mutant PRV and HSV-1 infections. In addition, PRV penetration through the BM was quite reduced by the loss of gE/gI and HSV-1 and BoHV-1 gE/gI null mutants even failed to cross the BM. Therefore, we conclude that the gE/gI complex is very important for the radial spread of alphaherpesviruses in the epithelium and crucial for their penetration through the BM (Fig. 5). In the veterinary world, safe vaccines, based on a gE deletion, have already been developed to protect against PRV and BoHV-1. The absence of gE attenuated the virus at the level of the respiratory mucosa (less and smaller plaques and reduced penetration through the BM and underlying connective tissue) and the level of retrograde transport along neurons. These marker vaccines successfully helped to eradicate PRV and BoHV-1 in multiple countries around the world (13, 21).

Finally, we demonstrated that the BoHV-1 gE/gI null mutant did not infect basal cells compared to PRV and HSV-1 mutants. It is very well possible that for this stage, there is a need for a protease. In addition, we found that the number of non-infected basal cells significantly increased underneath viral plaques and formed a triangular shape. The expression of integrinα6 was significantly upregulated underneath plaques, and its expression was redistributed from basolaterally toward the entire basal cell membrane. Under normal circumstances, the integrin α6 tightly connects basal cells to the BM, by binding laminin monomers (58). Upon infection, we hypothesized that basal cells reorganize integrin α6 expression to detach from the BM. Next, they migrate to and proliferate underneath viral plaques to form a wedge-like shape. The basal cell repair process has been well documented in the case of epithelial tissue damage (59). In the context of viral infections, a study recently demonstrated that SARS-CoV2 infection of polarized human airway epithelium induces a repair process that is sufficient in preventing viral infection of basal cells and basolateral shedding of the virus (60). We propose that this process may be part of an antiviral defense mechanism. Indeed, the gE/gI glycoprotein complex is known to downregulate the antiviral interferon (IFN) response (60). Interestingly, one of the functions of the interferon system in epithelia is to control the proliferation of basal cells (61–64). Therefore, future studies may compare IFN levels between WT and BoHV-1 gE/gI null mutant infection of respiratory explants.

**Fig 7:**
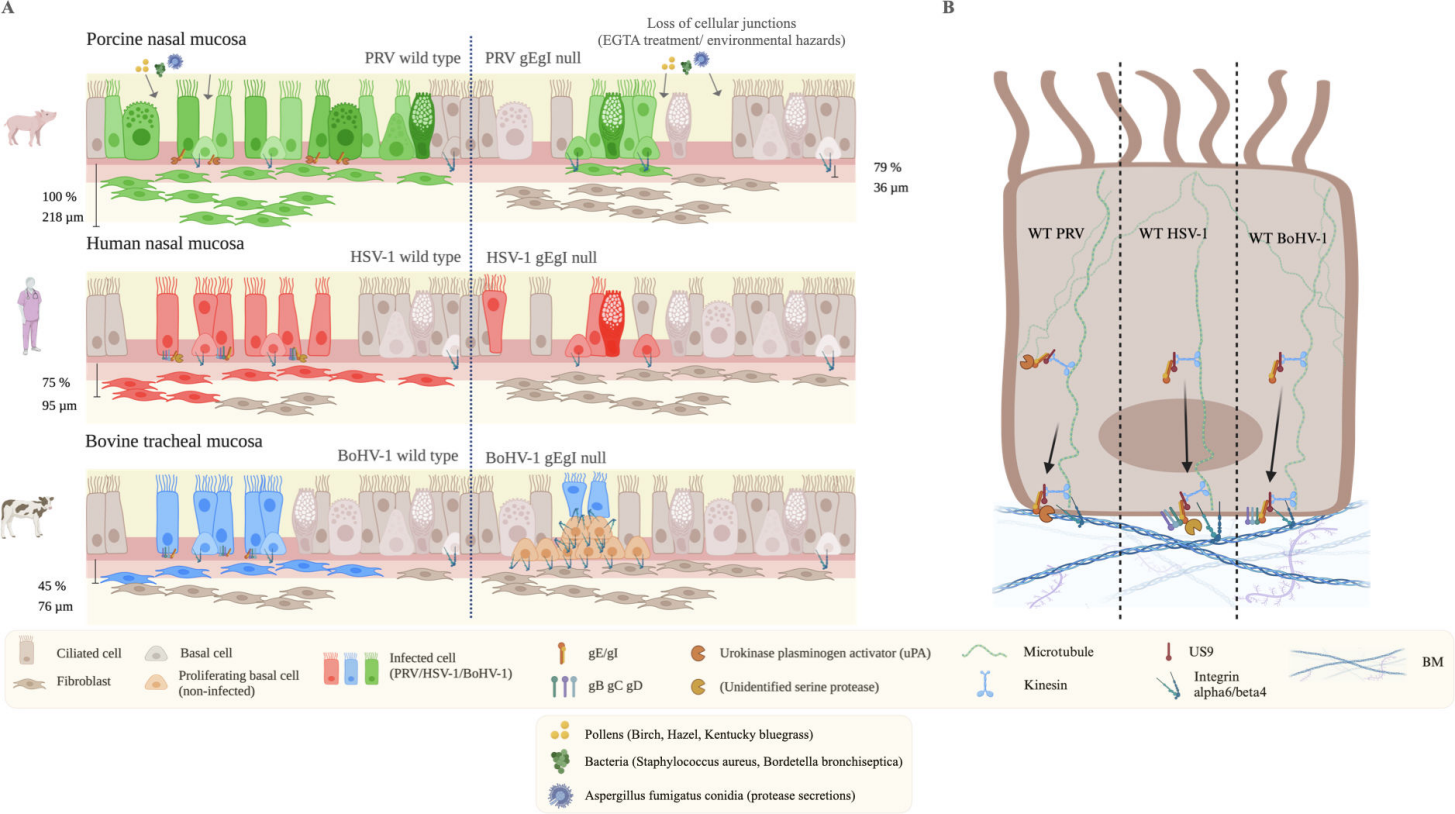
Comparative hypothetical model of alphaherpesvirus infection and invasion of the URT between species. (**A**) Situation at 72 hpi in compromised respiratory epithelium (caused by EGTA treatment or the action of environmental hazards). Top: PRV, middle: HSV-1, bottom: BoHV-1. Left of the dashed line: WT virus. Right of the dashed line: gE/gI null mutant virus. WT PRV infects faster and invades deeper the lamina propria than in HSV-1 and BoHV-1. The percentage of infection and plaque diameters are higher in PRV than HSV-1 and BoHV-1. PRV invasion goes up to 218 µm in 100% of the plaques. On the contrary, HSV-1 and BoHV-1 show less plaques with smaller plaque diameters. The percentage of plaques invading the BM and maximum invasion depths are lower as well (95 µm in 75% of plaques for HSV-1 and 76 µm in 45% of plaques for BoHV-1). Viral glycoproteins E, B, C, and D are more expressed at the BM by WT HSV-1 and WT BoHV-1 than by WT PRV. WT PRV invasion is facilitated by the urokinase plasminogen activator (uPA). HSV-1 invasion is also reliant upon a yet unidentified serine protease. No proteases are involved in BoHV-1 invasion. The glycoprotein E is crucial for BM invasion in all three viruses. Infection with gE/gI null mutants is both delayed and reduced in the three distinct epithelia compared to WT strains. Invasion of the BM is similarly reduced and delayed upon infection with gE/gI null viruses. Interestingly, BoHV-1 gE/gI null is additionally hampered in its invasion, as basal cells remain uninfected up to 72 hpi, suggesting that the gE/gI complex is necessary for basal cell infection in BoHV-1. Moreover, basal cells reorganize integrin α6 expression to detach from the BM. Next, they migrate to and proliferate underneath viral plaques to form a wedge-like shape. They push the infected cells away from the BM (sequestration process). (**B**) Hypothetical model explaining the different observed phenotypes. In all three WT viruses, the tail of the gE/gI complex binds to the US9 protein, which, in turn, binds to a kinesin motor protein. This kinesin specifically targets the basal side of the epithelial cell by moving along microtubuli. The extracellular domain of the gE/gI binds target proteins and traffics them to the basal side of the cell. In WT PRV, the urokinase plasminogen activator (uPA) is bound. In HSV-1, a yet unidentified serine protease is bound, as well as glycoproteins B, C, and D. In BoHV-1, no proteases are bound by the gE/gI complex. Only glycoproteins B, C, and D are bound and are transported to the basal side of the epithelial cells. Here, proteases facilitate invasion (WT PRV, WT HSV-1).

In conclusion, this study provides new insights into the infection and invasion of alphaherpesviruses in their host’s URT, with an emphasis on the interplay between the epithelial barrier system, protease activity, and the viral glycoprotein complex gE/gI.(Fig. 7)

## Data Availability

Raw data are available from the corresponding author upon reasonable request.

## SUPPLEMENTAL MATERIAL

Supplemental material part 1 mbio.01873-24-s0001.pdf Detailed explanations of materials and methods, as well as additional results and figures.

Supplemental material part 2 mbio.01873-24-s0002.pdf Detailed explanations of materials and methods, as well as additional results and a figure.

ASM does not own the copyrights to Supplemental Material that may be linked to, or accessed through, an article. The authors have granted ASM a non-exclusive, world-wide license to publish the Supplemental Material files. Please contact the corresponding author directly for reuse.
