# Supplementary material for: The alphaherpesvirus gE/gI glycoprotein complex and proteases jointly orchestrate invasion across the host’s upper respiratory epithelial barrier
Source: mBio. 2024 Oct 9;15(11):e01873-24. doi: 10.1128/mbio.01873-24 (PMC11558996; doi:10.1128/mbio.01873-24)
Supplement: Supplemental material part 1 — Detailed explanations of materials and methods, as well as additional results and figures. [file mbio.01873-24-s0001.pdf]

The Alphaherpesvirus gE/gI Glycoprotein Complex and Proteases Jointly Orchestrate Invasion Across the Host's Upper Respiratory Epithelial Barrier

Van Crombrugge E, Glorieux S, Zarak I, Van den Broeck W, Bachert C, Zhang N, Van Zele T, Smith GA, Laval K, Nauwynck H

Supplementary data part 1

Materials and methods

1. Calculation of the amount of glycoprotein expression at the basement membrane (BM)

Explants were infected with WT PRV, WT HSV-1 and WT BoHV-1 and fixed at 48 hpi. Double immunofluorescent stainings were performed to detect different main envelop proteins in the respiratory mucosa: glycoprotein E (gE), gB, gC and gD. Glycoprotein E was stained in green (Alexa Fluor 488), the other glycoproteins were stained in red (Alexa Fluor 594). To determine the amount of glycoprotein expression at the BM, the Image J (Fiji) software was used. Firstly, a region of interest (ROI) was manually drawn around the area of the basement membrane on the merged RGB picture. Afterwards, the channels were split into the different colors (green and red) and a threshold was determined to distinguish positive signal from noise. Next, the area fraction within the ROI that was positive for either green or red signal was determined using the *'measure'*→ *'area fraction'* function and represented as a percentage. The method is represented in Figure S1.
Similarly, HSV-1 and BoHV-1 gE/gI null infected explants were investigated for polarized glycoprotein expression. No expression of glycoprotein B or D (gC not determined, and gE not expressed) was found at the level of the BM (Figure S2).

2. Calculation of the number of basal cells underneath or next to plaque areas

Explants were infected with BoHV-1 gEgI null and fixed at 24, 48 and 72 hpi. Double immunofluorescent stainings were performed. BoHV-1 gC was stained in green (Alexa Fluor 488) to visualize viral plaques. Cytokeratin 15 (CK15) was stained in red (Alexa Fluor 594) to visualize basal cells. Different ROIs were determined. The first ROI (=ROI_1) was drawn

manually around and underneath a BoHV-1 gEgI null plaque. The dimentions of this ROI$_1$ were determined (i.e. area size, latitude, and height) and the ROI was saved in the '*ROI manager tool*'. Next, ROIs with the exact same dementions were drawn in a non-infected area in infected explants (=ROI$_{1'}$), or in a mock-inoculated explant (=ROI$_{1''}$). Afterwards, the number of epithelial cells were counted inside each ROI, followed by the number of CK15 positive cells. From these data, the percentage of basal cells in infected versus non infected areas was calculated.

3. Calculation of the percentage of integrin alpha 6 expression underneath or next to plaque areas

Determination of the different ROIs (ROI$_1$, ROI$_{1'}$ and ROI$_{1''}$) was performed in the exact same way as described above. Afterwards, within each ROI, the percentage of integrin alpha 6 was determined similarly to the calculation of the percentage of glycoprotein expression mentioned above (i.e. setting a threshold and determining the area fraction).

Figures

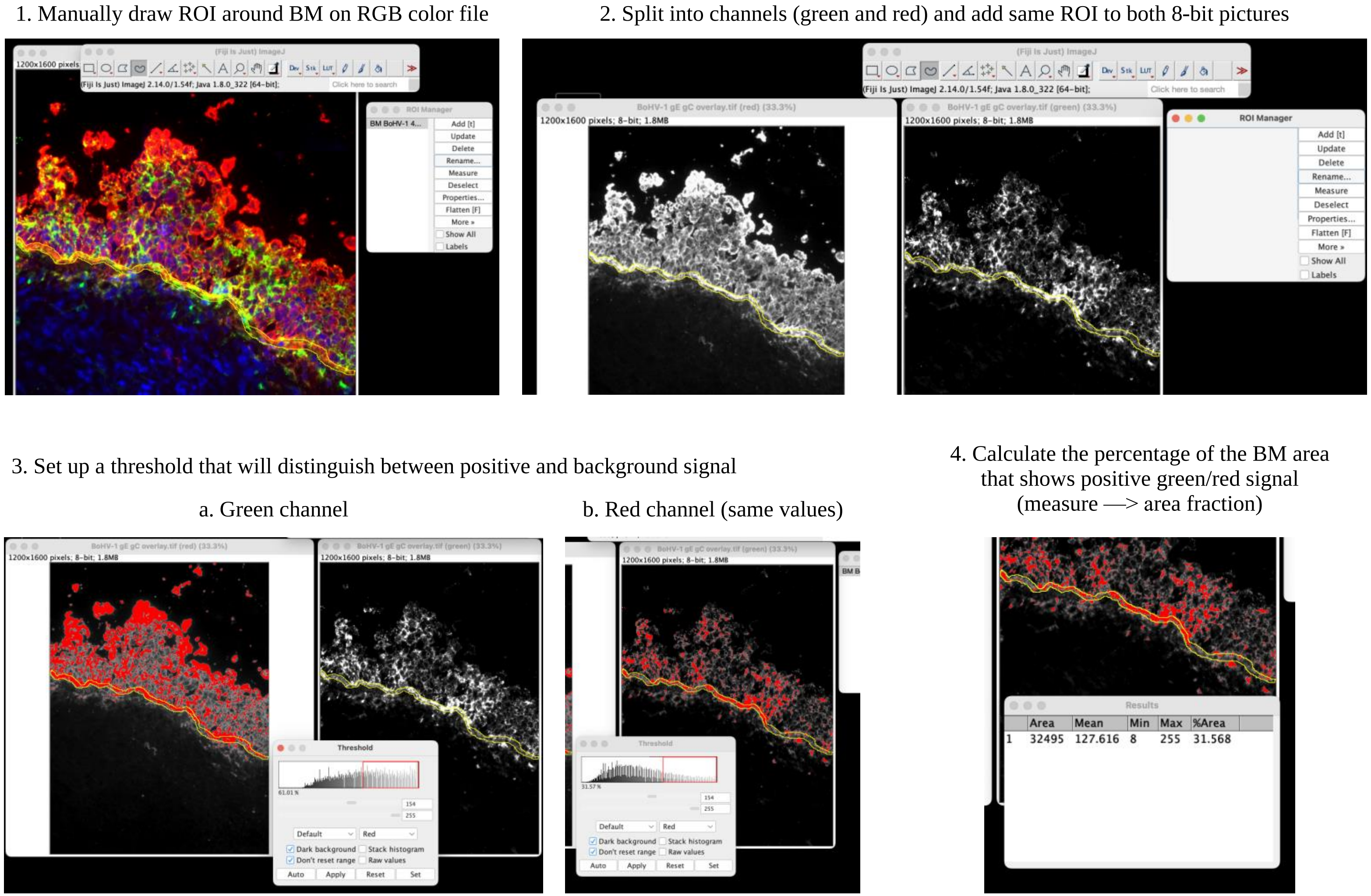

Figure S1. Example of the calculation method to determine glycoprotein expression at the BM

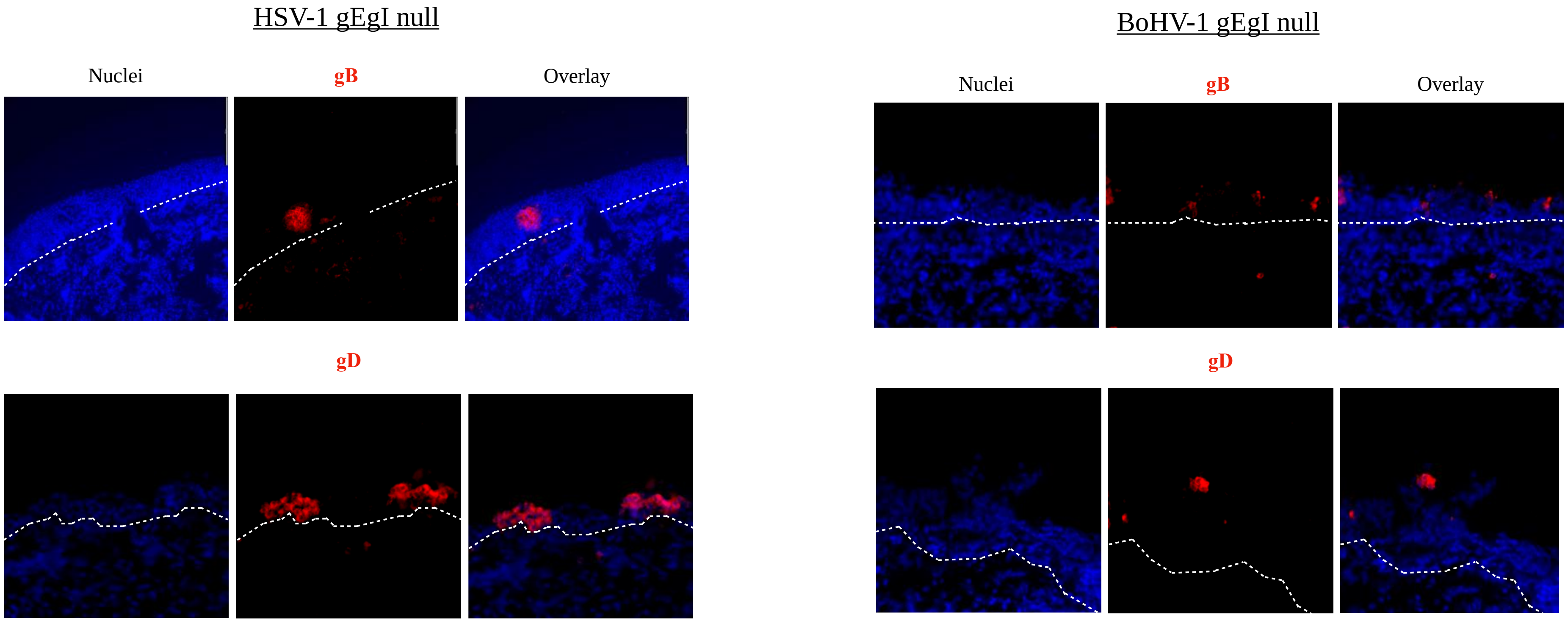

Figure S2. Expression of glycoproteins B and D in HSV-1 and BoHV-1 gE/gI null infection. Similary to the WT parental strains, the HSV-1 gE/gI null and BoHV-1 gE/gI null virus infected explants were stained to determine the polarization of the gB and gD glycoproteins. Contrary to the WT strains, there is no apparent polarization of gB or gD at the level of the basement membrane, suggesting that the gE/gI complex is responsible for the intracellular basolateral transport and deposition of these glycoproteins at the BM.
