# Supplementary material for: The alphaherpesvirus gE/gI glycoprotein complex and proteases jointly orchestrate invasion across the host’s upper respiratory epithelial barrier
Source: mBio. 2024 Oct 9;15(11):e01873-24. doi: 10.1128/mbio.01873-24 (PMC11558996; doi:10.1128/mbio.01873-24)
Supplement: Supplemental material part 2 — Detailed explanations of materials and methods, as well as additional results and a figure. [file mbio.01873-24-s0002.pdf]

The Alphaherpesvirus gE/gI Glycoprotein Complex and Proteases Jointly Orchestrate Invasion Across the Host's Upper Respiratory Epithelial Barrier

Van Crombrugge E, Glorieux S, Zarak I, Van den Broeck W, Bachert C, Zhang N, Van Zele T, Smith GA, Laval K, Nauwynck H

Supplementary data part 2

Materials and methods

4. Detection of urokinase plasminogen activator (uPA) in transfected HEK-T cells

HEK-T cells were plated on inserts in a 24-well plate and grown until 70% confluency before transfection. Human and bovine urokinase plasminogen activator cDNA clones (PLAU) in mammalian expression vectors (Genomics Online, Limerick PA, USA) were used. 500 ng of cDNA was diluted into opti-MEM medium (ThermoFisher) and transfected using a lipofectamine 3000 kit (ThermoFisher). 500 ng of cDNA was transfected per well. After 48 hours of incubation, HEK-T cells were fixed using 4% paraformaldehyde. Staining for uPa was performed with a rabbit polyclonal antibody directed against porcine uPA (ser21-pro168), followed by a goat-anti-rabbit IgG FITC secondary antibody. Figure S3 shows a positive detection of uPA in transfected HEK-T cell

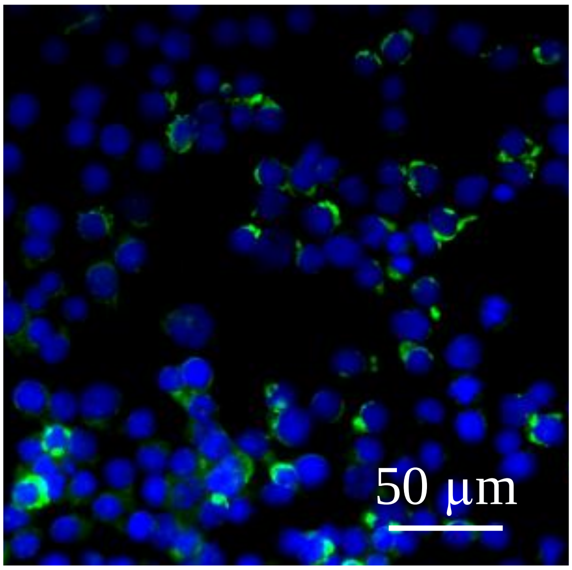

Figure S3. Detection of (human) urokinase plasminogen activator in transfected HEK-T cells. Cells were fixed at 48 hours post transfection. uPA was stained with a rabbit IgG polyclonal antibody directed against porcine uPA (ser21-pro168), followed by a goat-anti-rabbit IgG FITC secondary antibody (green color). Nuclei were counterstained with Hoechst 33342 (blue color).
